# 4-[2-(4-Chloro­phen­yl)hydrazinyl­idene]-3-methyl-1*H*-pyrazol-5(4*H*)-one

**DOI:** 10.1107/S1600536811037020

**Published:** 2011-09-17

**Authors:** Hoong-Kun Fun, Ching Kheng Quah, Nithinchandra, Balakrishna Kalluraya

**Affiliations:** a X-ray Crystallography Unit, School of Physics, Universiti Sains Malaysia, 11800 USM, Penang, Malaysia; b Department of Studies in Chemistry, Mangalore University, Mangalagangotri, Mangalore 574 199, India

## Abstract

In the title compound, C_10_H_9_ClN_4_O, the pyrazole ring [maximum deviation = 0.014 (2) Å] forms a dihedral angle of 7.06 (14)° with the chloro­benzene ring. The mol­ecular conformation is stabilized by an intra­molecular N—H⋯O hydrogen bond, which generates an *S*(6) ring motif. In the crystal, inversion dimers linked by pairs of C—H⋯O hydrogen bonds generate *R*_2_^2^(16) ring motifs. The dimers are further connected by N—H⋯N hydrogen bonds, thereby forming layers lying parallel to the *bc* plane.

## Related literature

For general background to and applications of pyrazole derivatives, see: Rai & Kalluraya (2006 ▶); Rai *et al.* (2008 ▶); Sridhar & Perumal (2003 ▶). For standard bond-length data, see: Allen *et al.* (1987 ▶). For graph-set notation, see: Bernstein *et al.* (1995 ▶). For the stability of the temperature controller used in the data collection, see: Cosier & Glazer (1986 ▶).
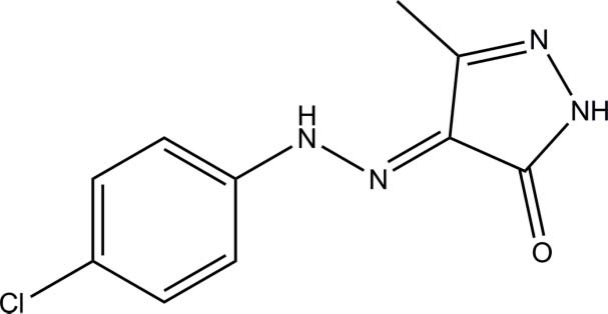


## Experimental

### 

#### Crystal data

C_10_H_9_ClN_4_O
*M* *_r_* = 236.66
Monoclinic, 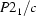

*a* = 15.8496 (5) Å
*b* = 3.8184 (1) Å
*c* = 20.3794 (6) Å
β = 123.575 (2)°
*V* = 1027.59 (5) Å^3^
*Z* = 4
Mo *K*α radiation
μ = 0.35 mm^−1^
*T* = 100 K
0.55 × 0.06 × 0.05 mm

#### Data collection

Bruker SMART APEXII CCD diffractometer
Absorption correction: multi-scan (*SADABS*; Bruker, 2009 ▶) *T*_min_ = 0.829, *T*_max_ = 0.984
11013 measured reflections
3048 independent reflections
2213 reflections with *I* > 2σ(*I*)
*R*_int_ = 0.050

#### Refinement

*R*[*F* ^2^ > 2σ(*F* ^2^)] = 0.063
*wR*(*F* ^2^) = 0.135
*S* = 1.08
3048 reflections
154 parameters
H atoms treated by a mixture of independent and constrained refinement
Δρ_max_ = 0.47 e Å^−3^
Δρ_min_ = −0.49 e Å^−3^

### 

Data collection: *APEX2* (Bruker, 2009 ▶); cell refinement: *SAINT* (Bruker, 2009 ▶); data reduction: *SAINT*; program(s) used to solve structure: *SHELXTL* (Sheldrick, 2008 ▶); program(s) used to refine structure: *SHELXTL*; molecular graphics: *SHELXTL*; software used to prepare material for publication: *SHELXTL* and *PLATON* (Spek, 2009 ▶).

## Supplementary Material

Crystal structure: contains datablock(s) global, I. DOI: 10.1107/S1600536811037020/hb6405sup1.cif

Structure factors: contains datablock(s) I. DOI: 10.1107/S1600536811037020/hb6405Isup2.hkl

Supplementary material file. DOI: 10.1107/S1600536811037020/hb6405Isup3.cml

Additional supplementary materials:  crystallographic information; 3D view; checkCIF report

## Figures and Tables

**Table 1:** Hydrogen-bond geometry (Å, °)

*D*—H⋯*A*	*D*—H	H⋯*A*	*D*⋯*A*	*D*—H⋯*A*
N1—H1*N*1⋯O1	0.93 (3)	2.15 (3)	2.841 (3)	131 (3)
N3—H1*N*3⋯N4^i^	0.87 (3)	2.16 (3)	2.983 (3)	158 (3)
C5—H5*A*⋯O1^ii^	0.95	2.47	3.334 (3)	151

Symmetry codes: (i) 
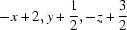
; (ii) 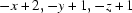.

**Fig. 1.**
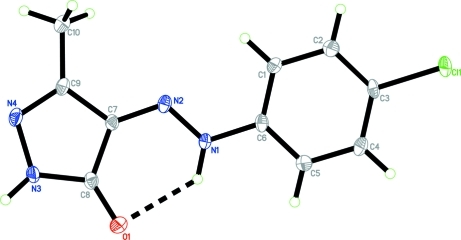
The molecular structure of the title compound showing 50% probability displacement ellipsoids for non-H atoms. The intramolecular hydrogen bond is shown as a dashed line.

**Fig. 2.**
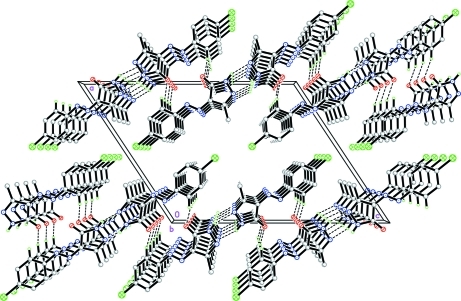
The crystal structure of the title compound, viewed along the b axis. H atoms not involved in hydrogen bonds (dashed lines) have been omitted for clarity.

## supplementary crystallographic information

### Comment

Pyrazole are nitrogen-containing heterocyclic compounds and various procedures have been developed for their synthesis (Rai & Kalluraya, 2006). The chemistry of pyrazole derivatives has been the subject of much interest due to their various applications and widespread potential and proven biological and pharmacological activities (Rai *et al.*, 2008). Steroids containing a pyrazole moiety are of interest as psychopharmacological agents. Some alkyl- and aryl-substituted pyrazoles have a sharply pronounced sedative action on the central nervous system. Furthermore, certain alkyl pyrazoles show significant bacteriostatic, bacteriocidal, fungicidal, analgesic and anti-pyretic activities (Sridhar & Perumal, 2003).

In the title molecule, Fig. 1, the pyrazole ring (N3/N4/C7-C9, maximum deviation of 0.014 (2) Å at atom N3) forms a dihedral angle of 7.06 (14)° with the phenyl ring (C1-C6). Bond lengths (Allen *et al.*, 1987) and angles are within normal ranges. The molecular structure is stabilized by an intramolecular N1–H1N1···O1 hydrogen bond, which generates an *S*(6) ring motif (Fig. 1, Bernstein *et al.*, 1995).

In the crystal, Fig. 2, the intermolecular C5–H5A···O1 hydrogen bonds (Table 1) form the inversion dimers producing sixteen-membered ring motifs R^2^_2_(16) (Bernstein *et al.*, 1995). Another intermolecular N3–H1N3···N4 hydrogen bond connects these dimers to another molecule forming two-dimensional layers parallel to *bc* plane.

### Experimental

To a solution of ethyl-(2-[2-(4-chlorophenyl)hydrazinylidene]-3-oxobutanoate (0.01 mol) dissolved in glacial acetic acid (20 ml), a solution of hydrazine hydrate (0.02 mol) in glacial acetic acid (25 ml) was added and the mixture was refluxed for 4 h. It is cooled and allowed to stand overnight. The solid product that separated was filtered and dried. It was then recrystallized from ethanol.  Yellow needles were obtained from 1:2 mixtures of DMF and ethanol by slow evaporation.

### Refinement

Atoms H1N1 and H3N3 were located from the difference Fourier map and refined freely [N–H = 0.87 (3) and  0.92 (3) Å]. The remaining H atoms were positioned geometrically and refined using a riding model with C–H = 0.95 or 0.98 Å and *U*_iso_(H) = 1.2 or 1.5 *U*_eq_(C). A rotating-group model was applied for the methyl group.

### Crystal data

**Table:**

C_10_H_9_ClN_4_O	*F*(000) = 488
*M**_r_* = 236.66	*D*_x_ = 1.530 Mg m^−^^3^
Monoclinic, *P*2_1_/*c*	Mo *K*α radiation, λ = 0.71073 Å
Hall symbol: -P 2ybc	Cell parameters from 3778 reflections
*a* = 15.8496 (5) Å	θ = 3.5–30.2°
*b* = 3.8184 (1) Å	µ = 0.35 mm^−^^1^
*c* = 20.3794 (6) Å	*T* = 100 K
β = 123.575 (2)°	Needle, yellow
*V* = 1027.59 (5) Å^3^	0.55 × 0.06 × 0.05 mm
*Z* = 4	

### Data collection

**Table:**

Bruker SMART APEXII CCD diffractometer	3048 independent reflections
Radiation source: fine-focus sealed tube	2213 reflections with *I* > 2σ(*I*)
graphite	*R*_int_ = 0.050
φ and ω scans	θ_max_ = 30.3°, θ_min_ = 2.0°
Absorption correction: multi-scan (*SADABS*; Bruker, 2009)	*h* = −22→22
*T*_min_ = 0.829, *T*_max_ = 0.984	*k* = −5→5
11013 measured reflections	*l* = −28→28

### Refinement

**Table:**

Refinement on *F*^2^	Primary atom site location: structure-invariant direct methods
Least-squares matrix: full	Secondary atom site location: difference Fourier map
*R*[*F*^2^ > 2σ(*F*^2^)] = 0.063	Hydrogen site location: inferred from neighbouring sites
*wR*(*F*^2^) = 0.135	H atoms treated by a mixture of independent and constrained refinement
*S* = 1.08	*w* = 1/[σ^2^(*F*_o_^2^) + (0.0465*P*)^2^ + 1.3049*P*] where *P* = (*F*_o_^2^ + 2*F*_c_^2^)/3
3048 reflections	(Δ/σ)_max_ = 0.001
154 parameters	Δρ_max_ = 0.47 e Å^−^^3^
0 restraints	Δρ_min_ = −0.49 e Å^−^^3^

### Special details

**Table:**

Experimental. The crystal was placed in the cold stream of an Oxford Cryosystems Cobra open-flow nitrogen cryostat (Cosier & Glazer, 1986) operating at 100.0 (1) K.
Geometry. All esds (except the esd in the dihedral angle between two l.s. planes) are estimated using the full covariance matrix. The cell esds are taken into account individually in the estimation of esds in distances, angles and torsion angles; correlations between esds in cell parameters are only used when they are defined by crystal symmetry. An approximate (isotropic) treatment of cell esds is used for estimating esds involving l.s. planes.
Refinement. Refinement of F^2^ against ALL reflections. The weighted R-factor wR and goodness of fit S are based on F^2^, conventional R-factors R are based on F, with F set to zero for negative F^2^. The threshold expression of F^2^ > 2sigma(F^2^) is used only for calculating R-factors(gt) etc. and is not relevant to the choice of reflections for refinement. R-factors based on F^2^ are statistically about twice as large as those based on F, and R- factors based on ALL data will be even larger.

### Fractional atomic coordinates and isotropic or equivalent isotropic displacement parameters (Å^2^)

**Table:**

	*x*	*y*	*z*	*U*_iso_*/*U*_eq_	
Cl1	0.53838 (4)	0.43205 (18)	0.09622 (3)	0.01992 (17)	
O1	1.00665 (12)	0.1519 (5)	0.58137 (9)	0.0189 (4)	
N1	0.81975 (15)	0.0862 (6)	0.43307 (10)	0.0142 (4)	
N2	0.78635 (14)	−0.0814 (6)	0.47044 (11)	0.0142 (4)	
N3	0.98057 (15)	−0.1246 (6)	0.67129 (11)	0.0161 (4)	
N4	0.90051 (14)	−0.2933 (6)	0.66973 (11)	0.0159 (4)	
C1	0.65048 (16)	0.0676 (7)	0.31307 (13)	0.0146 (5)	
H1A	0.6261	−0.0518	0.3403	0.018*	
C2	0.58548 (17)	0.1447 (7)	0.23340 (13)	0.0155 (5)	
H2A	0.5166	0.0740	0.2054	0.019*	
C3	0.62202 (17)	0.3249 (7)	0.19539 (12)	0.0142 (5)	
C4	0.72266 (17)	0.4294 (7)	0.23485 (13)	0.0157 (5)	
H4A	0.7465	0.5551	0.2079	0.019*	
C5	0.78795 (17)	0.3479 (6)	0.31411 (13)	0.0140 (5)	
H5A	0.8571	0.4155	0.3418	0.017*	
C6	0.75154 (17)	0.1671 (6)	0.35249 (12)	0.0135 (5)	
C7	0.85040 (16)	−0.1370 (6)	0.54667 (12)	0.0131 (5)	
C8	0.95460 (17)	−0.0130 (6)	0.59853 (12)	0.0140 (5)	
C9	0.82387 (17)	−0.3049 (7)	0.59611 (13)	0.0138 (5)	
C10	0.72429 (17)	−0.4604 (7)	0.57061 (14)	0.0171 (5)	
H10A	0.7304	−0.5904	0.6145	0.026*	
H10B	0.7027	−0.6200	0.5264	0.026*	
H10C	0.6741	−0.2735	0.5541	0.026*	
H1N1	0.887 (2)	0.154 (8)	0.4584 (16)	0.020 (7)*	
H1N3	1.029 (2)	−0.044 (9)	0.7166 (19)	0.033 (9)*	

### Atomic displacement parameters (Å^2^)

**Table:**

	*U*^11^	*U*^22^	*U*^33^	*U*^12^	*U*^13^	*U*^23^
Cl1	0.0212 (3)	0.0260 (3)	0.0096 (2)	0.0014 (3)	0.0067 (2)	0.0042 (2)
O1	0.0180 (8)	0.0234 (10)	0.0183 (8)	−0.0028 (7)	0.0120 (7)	−0.0005 (8)
N1	0.0153 (9)	0.0189 (10)	0.0096 (8)	−0.0012 (8)	0.0075 (7)	0.0008 (8)
N2	0.0189 (9)	0.0149 (9)	0.0122 (8)	0.0010 (8)	0.0107 (7)	−0.0007 (8)
N3	0.0148 (9)	0.0236 (11)	0.0100 (8)	−0.0009 (8)	0.0070 (8)	−0.0018 (8)
N4	0.0182 (9)	0.0203 (11)	0.0130 (8)	0.0026 (8)	0.0110 (8)	0.0009 (8)
C1	0.0163 (11)	0.0162 (11)	0.0143 (10)	−0.0002 (9)	0.0104 (9)	0.0008 (10)
C2	0.0163 (11)	0.0170 (12)	0.0144 (10)	0.0000 (9)	0.0093 (9)	−0.0021 (10)
C3	0.0179 (11)	0.0164 (11)	0.0068 (9)	0.0034 (9)	0.0059 (8)	0.0001 (9)
C4	0.0223 (11)	0.0152 (11)	0.0141 (10)	0.0007 (10)	0.0129 (9)	0.0011 (10)
C5	0.0156 (11)	0.0142 (11)	0.0138 (10)	−0.0005 (9)	0.0091 (9)	−0.0026 (9)
C6	0.0185 (11)	0.0137 (11)	0.0096 (9)	0.0017 (9)	0.0085 (8)	−0.0013 (9)
C7	0.0135 (10)	0.0164 (12)	0.0106 (9)	0.0009 (9)	0.0075 (8)	−0.0012 (9)
C8	0.0160 (11)	0.0159 (12)	0.0120 (9)	0.0015 (9)	0.0088 (8)	−0.0019 (9)
C9	0.0184 (11)	0.0132 (11)	0.0136 (10)	0.0026 (9)	0.0111 (9)	0.0013 (9)
C10	0.0201 (11)	0.0171 (12)	0.0182 (10)	0.0006 (10)	0.0131 (9)	0.0017 (10)

### Geometric parameters (Å, °)

**Table:**

Cl1—C3	1.745 (2)	C2—C3	1.380 (3)
O1—C8	1.232 (3)	C2—H2A	0.9500
N1—N2	1.310 (3)	C3—C4	1.390 (3)
N1—C6	1.413 (3)	C4—C5	1.389 (3)
N1—H1N1	0.92 (3)	C4—H4A	0.9500
N2—C7	1.321 (3)	C5—C6	1.386 (3)
N3—C8	1.369 (3)	C5—H5A	0.9500
N3—N4	1.407 (3)	C7—C9	1.439 (3)
N3—H1N3	0.87 (3)	C7—C8	1.462 (3)
N4—C9	1.309 (3)	C9—C10	1.486 (3)
C1—C6	1.390 (3)	C10—H10A	0.9800
C1—C2	1.391 (3)	C10—H10B	0.9800
C1—H1A	0.9500	C10—H10C	0.9800
			
N2—N1—C6	119.14 (19)	C6—C5—H5A	120.2
N2—N1—H1N1	121.3 (17)	C4—C5—H5A	120.2
C6—N1—H1N1	119.5 (17)	C5—C6—C1	121.1 (2)
N1—N2—C7	117.88 (19)	C5—C6—N1	118.4 (2)
C8—N3—N4	113.00 (18)	C1—C6—N1	120.6 (2)
C8—N3—H1N3	127 (2)	N2—C7—C9	124.3 (2)
N4—N3—H1N3	116 (2)	N2—C7—C8	128.6 (2)
C9—N4—N3	107.15 (19)	C9—C7—C8	106.78 (18)
C6—C1—C2	119.3 (2)	O1—C8—N3	128.2 (2)
C6—C1—H1A	120.3	O1—C8—C7	128.8 (2)
C2—C1—H1A	120.3	N3—C8—C7	102.99 (19)
C3—C2—C1	119.4 (2)	N4—C9—C7	110.0 (2)
C3—C2—H2A	120.3	N4—C9—C10	123.2 (2)
C1—C2—H2A	120.3	C7—C9—C10	126.8 (2)
C2—C3—C4	121.5 (2)	C9—C10—H10A	109.5
C2—C3—Cl1	118.75 (17)	C9—C10—H10B	109.5
C4—C3—Cl1	119.76 (18)	H10A—C10—H10B	109.5
C5—C4—C3	119.2 (2)	C9—C10—H10C	109.5
C5—C4—H4A	120.4	H10A—C10—H10C	109.5
C3—C4—H4A	120.4	H10B—C10—H10C	109.5
C6—C5—C4	119.5 (2)		
			
C6—N1—N2—C7	−176.4 (2)	N1—N2—C7—C9	177.5 (2)
C8—N3—N4—C9	2.5 (3)	N1—N2—C7—C8	5.1 (4)
C6—C1—C2—C3	−1.4 (4)	N4—N3—C8—O1	177.3 (2)
C1—C2—C3—C4	0.4 (4)	N4—N3—C8—C7	−2.6 (3)
C1—C2—C3—Cl1	−177.90 (19)	N2—C7—C8—O1	−4.7 (4)
C2—C3—C4—C5	0.5 (4)	C9—C7—C8—O1	−178.2 (2)
Cl1—C3—C4—C5	178.85 (19)	N2—C7—C8—N3	175.2 (2)
C3—C4—C5—C6	−0.6 (4)	C9—C7—C8—N3	1.8 (3)
C4—C5—C6—C1	−0.4 (4)	N3—N4—C9—C7	−1.2 (3)
C4—C5—C6—N1	179.7 (2)	N3—N4—C9—C10	−179.2 (2)
C2—C1—C6—C5	1.4 (4)	N2—C7—C9—N4	−174.2 (2)
C2—C1—C6—N1	−178.8 (2)	C8—C7—C9—N4	−0.4 (3)
N2—N1—C6—C5	179.3 (2)	N2—C7—C9—C10	3.7 (4)
N2—N1—C6—C1	−0.6 (3)	C8—C7—C9—C10	177.5 (2)

### Hydrogen-bond geometry (Å, °)

**Table:**

*D*—H···*A*	*D*—H	H···*A*	*D*···*A*	*D*—H···*A*
N1—H1N1···O1	0.93 (3)	2.15 (3)	2.841 (3)	131 (3)
N3—H1N3···N4^i^	0.87 (3)	2.16 (3)	2.983 (3)	158 (3)
C5—H5A···O1^ii^	0.95	2.47	3.334 (3)	151

Symmetry codes: (i) −*x*+2, *y*+1/2, −*z*+3/2; (ii) −*x*+2, −*y*+1, −*z*+1.
